# Leveraging machine learning to distinguish between bacterial and viral induced pharyngitis using hematological markers: a retrospective cohort study

**DOI:** 10.1038/s41598-023-49925-1

**Published:** 2023-12-21

**Authors:** Zhe Jin, Fengmei Ma, Haoyang Chen, Shufan Guo

**Affiliations:** 1 School of Medical Technology, Hebei Medical University, Shijiazhuang, 050017 People’s Republic of China; 2 Department of Otorhinolaryngology, Hebei Provincial Hospital of Traditional Chinese Medicine, Shijiazhuang, 050011 People’s Republic of China; 3 Medicine-Education Coordination and Medical Education Research Center, Hebei Medical University, Shijiazhuang, 050017 People’s Republic of China

**Keywords:** Biomarkers, Diagnosis, Public health

## Abstract

Accurate differentiation between bacterial and viral-induced pharyngitis is recognized as essential for personalized treatment and judicious antibiotic use. From a cohort of 693 patients with pharyngitis, data from 197 individuals clearly diagnosed with bacterial or viral infections were meticulously analyzed in this study. By integrating detailed hematological insights with several machine learning algorithms, including Random Forest, Neural Networks, Decision Trees, Support Vector Machine, Naive Bayes, and Lasso Regression, for potential biomarkers were identified, with an emphasis being placed on the diagnostic significance of the Monocyte-to-Lymphocyte Ratio. Distinct inflammatory signatures associated with bacterial infections were spotlighted in this study. An innovation introduced in this research was the adaptation of the high-accuracy Lasso Regression model for the TI-84 calculator, with an AUC (95% CI) of 0.94 (0.925–0.955) being achieved. Using this adaptation, pivotal laboratory parameters can be input on-the-spot and infection probabilities can be computed subsequently. This methodology embodies an improvement in diagnostics, facilitating more effective distinction between bacterial and viral infections while fostering judicious antibiotic use.

## Introduction

Pharyngitis, defined by pharyngeal inflammation, is a predominant concern within the department of otorhinolaryngology, affecting a vast number of individuals annually^1^. Plenty of infectious agents can induce pharyngitis; however, bacterial sources, especially Group A β-hemolytic streptococcus (GABHS) is most prevalent^2^. Concurrently, viral infections continue to present significant clinical complexities^3^. Accurately differentiating between bacterial and viral pharyngitis is critical not only for precise therapeutic strategies but also to curb the overuse of antibiotics, a trend exacerbating the global rise of antibiotic-resistant organisms^4,5^.

In the past decade, the potential of complete blood count (CBC) parameters such as Neutrophil-to-Lymphocyte ratio (NLR) and Monocyte-to-Lymphocyte ratio (MLR) has been extensively explored as biomarkers for early diagnosis in cancers^6–9^. These markers have also shown promise in differentiating between bacterial and viral infections, providing a non-invasive, cost-effective approach to aid in clinical decision-making^10,11^. Nonetheless, the differentiation between viral and bacterial pharyngitis still poses a significant challenge, calling for more advanced and precise diagnostic tools^12,13^.

Recently, machine learning (ML) has gained traction in healthcare for its potential to revolutionize diagnosis and treatment^14,15^. ML models, capable of learning from large datasets and identifying complex patterns, have shown promise in infections. Despite these advances, there is still a paucity of research exploring the utility of machine learning in differentiating between bacterial and viral pharyngitis specifically^16,17^.

Therefore, this study aims to develop and validate an ML model specifically tailored to distinguish between viral and bacterial pharyngitis, improving diagnosis accuracy^18^ and promoting more responsible antibiotic stewardship^19,20^.

## Methods

### Study design

This retrospective cohort study included adult patients with pharyngitis caused by different infection types. An evaluation of the diagnostic accuracy of bacterial and viral pharyngitis across various demographic groups was conducted based on a retrospective study design. A comprehensive clinical examination has been undertaken for each participant, considering their medical history and current symptoms.

### Patients’ recruitment

The patients with pharyngitis were enrolled in the study through a systematic recruitment process that aimed to ensure the inclusion of eligible participants with complete and relevant data. The recruitment process followed several steps to identify and select suitable candidates:

1. Patient Identification: Potential participants with symptoms of pharyngitis were identified from the patient population attending the Department of Otorhinolaryngology at Hebei Provincial Hospital of Traditional Chinese Medicine, between 2019 and 2023.
2. Screening for patients with pharyngitis: This screening involved a review of their medical history and a clinical examination.
3. Inclusion Criteria: To be included in the study, patients had to meet the following criteria:
  3.1. Confirmed diagnosis of pharyngitis based on clinical evaluation.
  3.2. Absence of severe medical conditions or comorbidities that could confound the analysis.
4. Exclusion Criteria: Patients with the following characteristics were excluded from the study:
  4.1. Absence of essential demographic details or incomplete data pertaining to complete cell count metrics.
  4.2. Age below 18 years.
  4.3. Patients without a definitive diagnosis of infection type.

### Independent variables

Several parameters, including basic demographic information, complete cell count, and novel parameters such as the NLR, platelet-to-lymphocyte ratio (PLR), monocyte-to-lymphocyte ratio (MLR), and mean platelet volume-to-lymphocyte ratio (MPVLR), were assessed to provide a comprehensive picture. These novel parameters have been log-transformed prior to analysis to manage skewness, stabilize variance, lessen the influence of outliers, and convert multiplicative relationships into more interpretable additive ones, enhancing the robustness and validity of our statistical tests.

### Statistical analysis

The current investigation employed a dataset comprising diverse clinical metrics, indicative of either bacterial or viral infections. To ensure a balanced comparison between the different infection types, a 1:1 propensity score matching (PSM) was utilized. Following this matching, the dataset was randomly partitioned into a training cohort (75%) and a validation cohort (25%). Continuous variables were evaluated using two-sample t-tests, while categorical variables were assessed through chi-squared tests. The threshold for statistical significance was set at *p* = 0.05. Analytical computations were conducted using R (version 3.6.3) and Python (version 3.7).

### Machine learning analysis

A suite of machine learning algorithms was applied to selected clinical parameters to develop predictive models. The algorithms employed included Lasso Regression, Decision Trees, Random Forest, Support Vector Machines (SVM), Neural Networks (NN), and Naive Bayes (NB). The performance of these algorithms was evaluated using metrics such as accuracy, precision, recall, F1-score, and the area under the Receiver Operating Characteristic (AUC) curve. Furthermore, the importance of each feature was assessed across various models to ascertain their contribution to the predictive power of the models.

Visual representations, including ROC curves, were crafted for each model to enable a comparative evaluation of their performances between training and validation cohorts. Additionally, violin plots illustrated the distribution of clinical metrics across the two infection types.

### Model deployment

The Lasso regression model was encapsulated into a TI-84 calculator via a custom script, engineered for rapid input of laboratory parameters. Upon input, an output delineating the infection type probability was generated. The model's performance was stringently evaluated and validated using our designated validation cohort.

### Ethical compliance

This study is approved by the Medical Ethical Committee of Hebei Provincial Hospital of Traditional Chinese Medicine, the register num is HBZY2023-KY-012-01. A waiver for the requirement of informed consent has been granted by the Medical Ethical Committee of Hebei Provincial Hospital of Traditional Chinese Medicine due to its retrospective nature. Strict adherence to the ethical guidelines related to human subjects in research was maintained in our study. all their privacy and confidentiality were upheld throughout the study.

## Results

**Figure 1:**
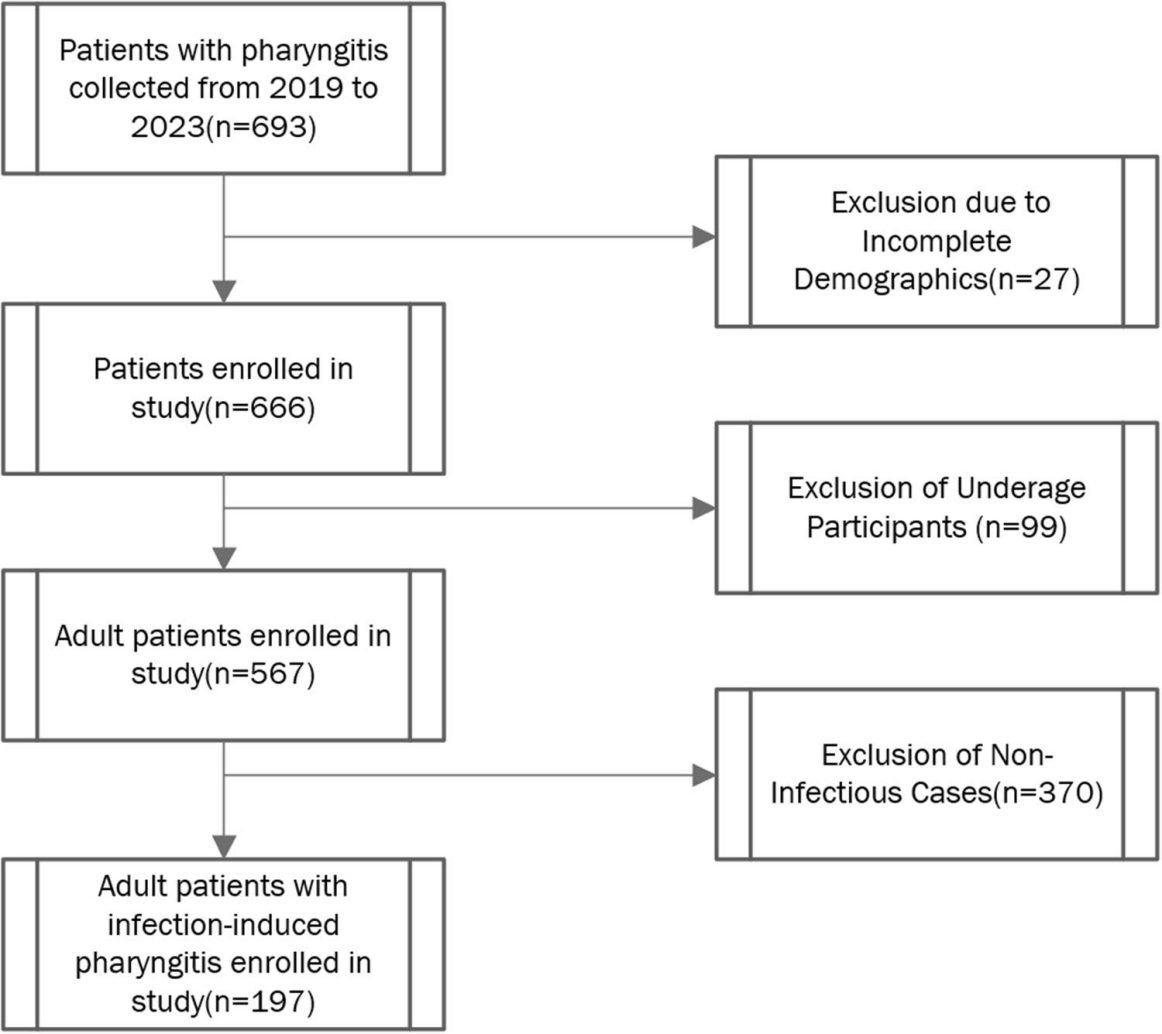
Flowchart of the study design and patient categorization. A comprehensive flowchart illustrating the data collection and selection process is provided.

A total of 693 patients diagnosed with pharyngitis were initially identified. Following rigorous adherence to predefined inclusion and exclusion criteria, a cohort of 197 eligible patients was delineated. This cohort included 74 individuals diagnosed with viral infections and 123 with bacterial infections, as depicted in Fig. 1. These participants were then methodically allocated into two primary cohorts for further analysis: the training cohort, consisting of 55 individuals with bacterial infections and 56 with viral infections, and the validation cohort, comprising 19 individuals with bacterial infections and 18 with viral infections. This stratification provided a structured framework for the comparative analysis of viral and bacterial pharyngitis cases, thereby facilitating the subsequent development and validation of machine learning models.

Demographic attributes such as sex, age, and clinical status (outpatient or inpatient) were recorded. An approximately balanced distribution of males and females was observed across both cohorts, aligning with the demographic findings in related literature^21^. Age ranged from 18 to 85 years, with the most represented age group being 18–34 years. Majority of the patients were outpatients, with no significant difference in distribution between the two infection types (Table 1).

**Table 1:** Comparative analysis of patient characteristics and hematological indices between patients with viral and bacterial induced pharyngitis.

Characteristics	Viral infection (n = 74)	Bacterial infection (n = 123)	*p* value
Sex, n (%)			
Male	35 (17.8%)	57 (28.9%)	0.896
Female	39 (19.8%)	66 (33.5%)	
AgeGroup, n (%)			
18 ~ 34y	48 (24.4%)	57 (28.9%)	0.058
35 ~ 54y	14 (7.1%)	41 (20.8%)	
55 ~ 64y	7 (3.6%)	8 (4.1%)	
65 ~ 74y	3 (1.5%)	14 (7.1%)	
75 ~ 84y	2 (1%)	2 (1%)	
≥ 85y	0 (0%)	1 (0.5%)	
Type, n (%)			
Outpatient	70 (35.5%)	115 (58.4%)	0.996
Inpatient	4 (2%)	8 (4.1%)	
RBC, median (IQR)	4.945 (4.4575, 5.2575)	4.57 (4.335, 5.03)	0.003
HGB, mean ± sd	147.26 ± 21.22	139.37 ± 15.293	0.006
WBC, median (IQR)	8.6 (4.33, 10.652)	10.33 (8.63, 11.84)	< 0.01
NEU, median (IQR)	4.81 (2.115, 6.445)	7.47 (6.375, 9.085)	< 0.01
NEUp, mean ± sd	56.484 ± 13.455	76.372 ± 6.8947	< 0.01
MONO, median (IQR)	0.485 (0.3225, 0.57)	0.6 (0.41, 0.75)	< 0.01
MONOp, median (IQR)	5.95 (4.925, 7.05)	5.6 (4.65, 6.65)	0.120
LYM, median (IQR)	2.415 (1.55, 3.485)	1.73 (1.12, 2.155)	< 0.01
LYMp, median (IQR)	33.4 (26.7, 39.55)	16.2 (12.7, 19.85)	< 0.01
PLT, median (IQR)	247 (201, 299)	263 (207.5, 307.5)	0.320
MPV, median (IQR)	9.1 (8.5, 9.8)	8.9 (8.5, 9.5)	0.094
logMLR, median (IQR)	− 1.6938 (− 1.9686, − 1.3214)	− 1.0525 (− 1.2915, − 0.79727)	< 0.01
logNLR, mean ± sd	0.53354 ± 0.67993	1.576 ± 0.43285	< 0.01
logPLR, mean ± sd	4.6365 ± 0.56854	5.0804 ± 0.41432	< 0.01
logMPVLR, median (IQR)	1.2873 (0.92734, 1.814)	1.6546 (1.4603, 2.022)	< 0.01

Lists the demographic and hematological parameters studied. Data are presented as n (%) for categorical variables, median (Interquartile Range, IQR) for non-normally distributed continuous variables, and mean ± standard deviation (sd) for normally distributed continuous variables. The standards of error analysis and ranges have been accounted for in the provided IQR and sd.

Outcome measures focused on hematological indices. The observed variations included higher Red blood cell (RBC) count and Hemoglobin concentration (HGB) levels in patients with viral infections (*p* = 0.003 and *p* = 0.006, respectively) and elevated White Blood Cell (WBC) count, Neutrophil count (NEU) count, and Monocyte count (MONO) count in patients with bacterial infections (*p* < 0.01 for each parameter). Significant differences were noted for other parameters such as Percentage of Neutrophils (NEUp), Lymphocyte (LYM) count, Percentage of Lymphocytes (LYMp), log-transformed Monocyte to Lymphocyte ratio (logMLR), log-transformed Neutrophil to Lymphocyte ratio (logNLR), and log-transformed Platelet to Lymphocyte ratio (logPLR) between the two infection groups (Table 1).

A comparative analysis revealed significant differences in several hematological indices between the viral and bacterial infection groups in both the training and validation cohorts. Notably, in the training cohort, there were significant variations regarding HGB, WBC, NEU, NEUp, LYM, LYMp, logMLR, logNLR, logPLR, and log-transformed Platelet Volume to Lymphocyte ratio (logMPVLR) (all *p* < 0.05). Meanwhile, the validation cohort displayed significant differences for NEU, NEUp, LYMp, logMLR, and logNLR (all *p* < 0.05) (Table 2). These findings echo the inherent diagnostic challenges associated with pharyngitis, where overlapping symptoms between bacterial, primarily caused by Group A β-hemolytic streptococcus, and viral pharyngitis often complicate accurate diagnosis^22^. Although blood tests have been instrumental in aiding the diagnosis of acute viral and bacterial infections, their efficacy is sometimes hindered by their inability to capture the evolving inflammatory response post-symptom onset^23^.

**Table 2:** Hematological parameters in bacterial and viral infections in training and validation cohorts.

Characteristics	Training cohort			Validation cohort		
	Bacterial infection (n = 55)	Viral infection (n = 56)	*p* value	Bacterial infection (n = 19)	Viral infection (n = 18)	*p* value
Sex, n (%)						
Female	31 (27.9%)	31 (27.9%)	0.915	10 (27%)	7 (18.9%)	0.515
Male	24 (21.6%)	25 (22.5%)		9 (24.3%)	11 (29.7%)	
AgeGroup, n (%)						
18 ~ 34y	32 (28.8%)	25 (22.5%)	0.590	11 (29.7%)	10 (27%)	1.000
35 ~ 54y	13 (11.7%)	20 (18%)		6 (16.2%)	7 (18.9%)	
65 ~ 74y	5 (4.5%)	7 (6.3%)		1 (2.7%)	0 (0%)	
55 ~ 64y	4 (3.6%)	3 (2.7%)		1 (2.7%)	0 (0%)	
75 ~ 84y	1 (0.9%)	1 (0.9%)		0 (0%)	1 (2.7%)	
Type, n (%)						
Outpatient	51 (45.9%)	53 (47.7%)	0.980	17 (45.9%)	17 (45.9%)	1.000
Inpatient	4 (3.6%)	3 (2.7%)		2 (5.4%)	1 (2.7%)	
RBC, mean ± sd	4.6602 ± 0.59088	4.8616 ± 0.547	0.065	4.6 (4.365, 4.9)	5.23 (4.7725, 5.555)	0.053
HGB, mean ± sd	138.44 ± 16.049	146.88 ± 19.301	0.014	137.95 ± 13.571	147.28 ± 26.479	0.193
WBC, median (IQR)	10 (8.415, 11.52)	9.04 (4.935, 10.58)	0.017	9.37 (7.93, 11.085)	9.755 (3.6825, 11.232)	0.533
NEU, median (IQR)	7.46 (6.35, 8.355)	4.94 (2, 6.51)	< 0.01	6.91 (6.3, 8.08)	6.09 (2.1325, 6.955)	0.018
NEUp, median (IQR)	76.3 (73, 81.45)	56.85 (51.175, 62.25)	< 0.01	77.779 ± 6.4076	59.372 ± 13.63	< 0.01
MONO, median (IQR)	0.55 (0.375, 0.63)	0.455 (0.3175, 0.57)	0.137	0.49579 ± 0.20815	0.48 ± 0.21647	0.822
MONOp, median (IQR)	5.3 (4.2, 6.45)	5.75 (4.85, 6.95)	0.053	5.2 (4.3, 6.5)	6.2 (5.15, 6.55)	0.201
LYM, median (IQR)	1.66 (1.015, 2.065)	3.08 (1.8425, 3.475)	< 0.01	1.45 (1.02, 2.07)	2.065 (1.195, 3.495)	0.086
LYMp, median (IQR)	16.2 (12.05, 19)	34.5 (29.4, 39.575)	< 0.01	15.826 ± 5.4765	31.461 ± 13.564	< 0.01
PLT, mean ± sd	258.4 ± 64.522	259.05 ± 75.76	0.961	262.37 ± 43.69	245.28 ± 75.568	0.410
MPV, median (IQR)	9 (8.5, 9.5)	9.1 (8.6, 9.8)	0.224	8.9316 ± 0.70636	8.95 ± 0.92498	0.946
logMLR, median (IQR)	-1.0782 (-1.3508, -0.84269)	-1.8133 (-1.9973, -1.3744)	< 0.01	− 1.1307 ± 0.44905	− 1.5568 ± 0.45629	< 0.01
logNLR, median (IQR)	1.5529 (1.316, 1.8971)	0.50511 (0.28874, 0.75537)	< 0.01	1.6467 ± 0.43002	0.69269 ± 0.68404	< 0.01
logPLR, mean ± sd	5.1281 ± 0.42824	4.585 ± 0.51646	< 0.01	5.2478 ± 0.44374	4.7693 ± 0.73847	0.022
logMPVLR, mean ± sd	1.8156 ± 0.48449	1.3021 ± 0.55645	< 0.01	1.8786 ± 0.49013	1.4979 ± 0.6658	0.055

This table summarizes the hematological parameters and their logarithmically transformed ratios for both bacterial and viral infections in the training and validation cohorts. Variables include red blood cell count (RBC), hemoglobin (HGB), white blood cell count (WBC), neutrophils (NEU), monocytes (MONO), lymphocytes (LYM), platelets (PLT), mean platelet volume (MPV), monocyte to lymphocyte ratio (logMLR), neutrophil to lymphocyte ratio (logNLR), platelet to lymphocyte ratio (logPLR), and mean platelet volume to lymphocyte ratio (logMPVLR). Significance levels (*p*-values) are reported for each variable, with < 0.05 considered significant.

**Figure 2:**
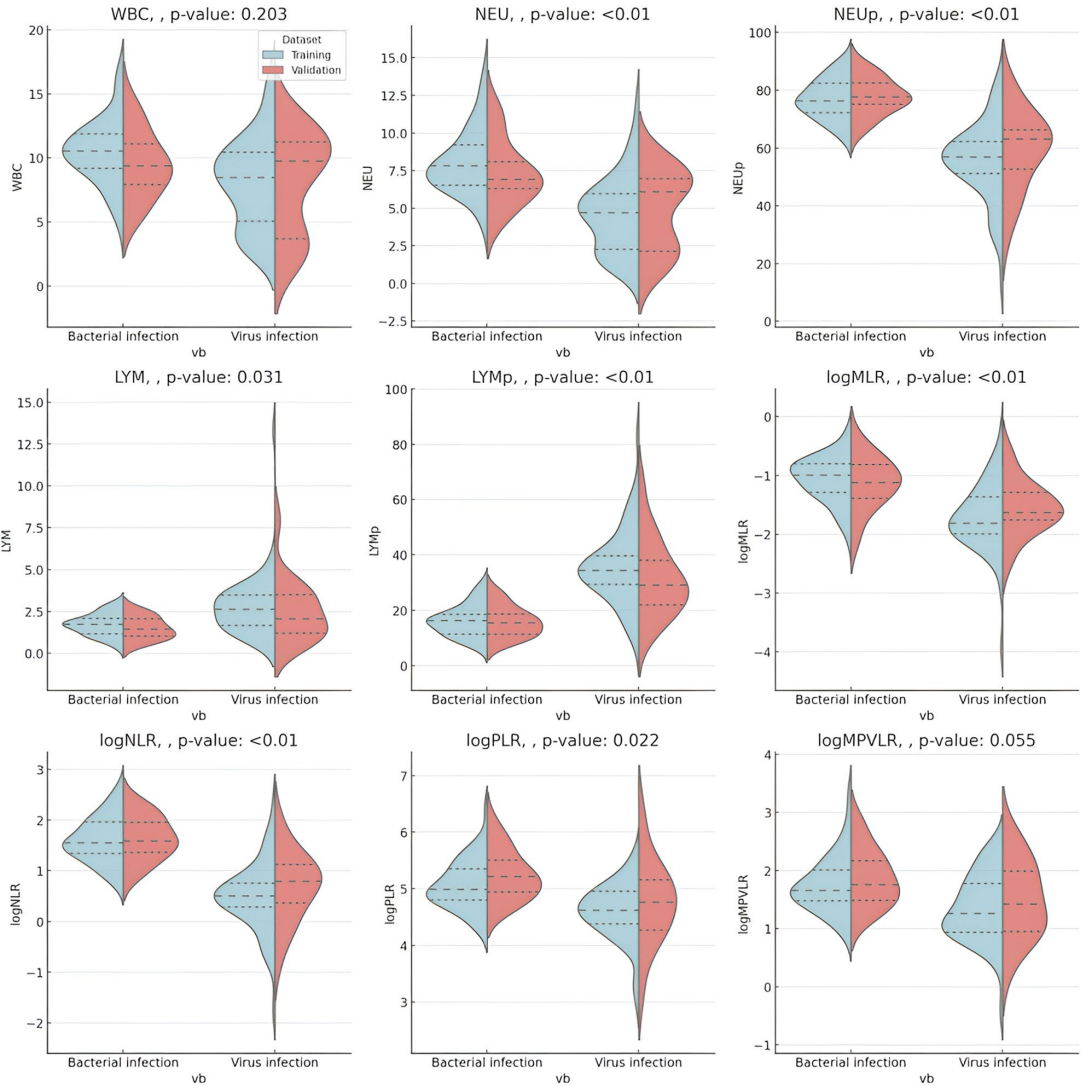
Distribution of Hematological and Inflammatory Parameters Amid Bacterial and Viral Infections. The violin plots showcase the distribution of several hematological and inflammatory parameters including 'WBC' (white blood cell count), 'NEU' (neutrophils), 'NEUp' (neutrophil percentage), 'MONO' (monocytes), 'LYM' (lymphocytes), 'LYMp' (lymphocyte percentage), 'logMLR' (log-transformed monocyte-to-lymphocyte ratio), 'logNLR' (log-transformed neutrophil-to-lymphocyte ratio), and 'logPLR' (log-transformed platelet-to-lymphocyte ratio) in cases of bacterial and viral infections. Each violin depicts the density distribution of the data, with the width indicating data density. The white dot represents the median, the thick bar illustrates the interquartile range, and the thin line encompasses the remaining data distribution, excluding outliers determined by a function of the interquartile range. These plots elucidate distinct trends between the two infection types.

The violin plots demonstrated distinct trends in hematological and inflammatory parameters between bacterial and viral infections. Parameters such as WBC and MONO had overlapping distributions, while NEU, NEUp, logMLR, logNLR, and logPLR were predominantly higher in bacterial infections. LYM and LYMp leaned more towards viral infections (Fig. 2). It was observed that parameters like WBC count and MONO count exhibited overlapping distributions, hinting at a common inflammatory response irrespective of the infection type. Conversely, parameters such as NEU, NEUp, logMLR, logNLR, and logPLR demonstrated elevated levels predominantly in bacterial infections.

**Figure 3:**
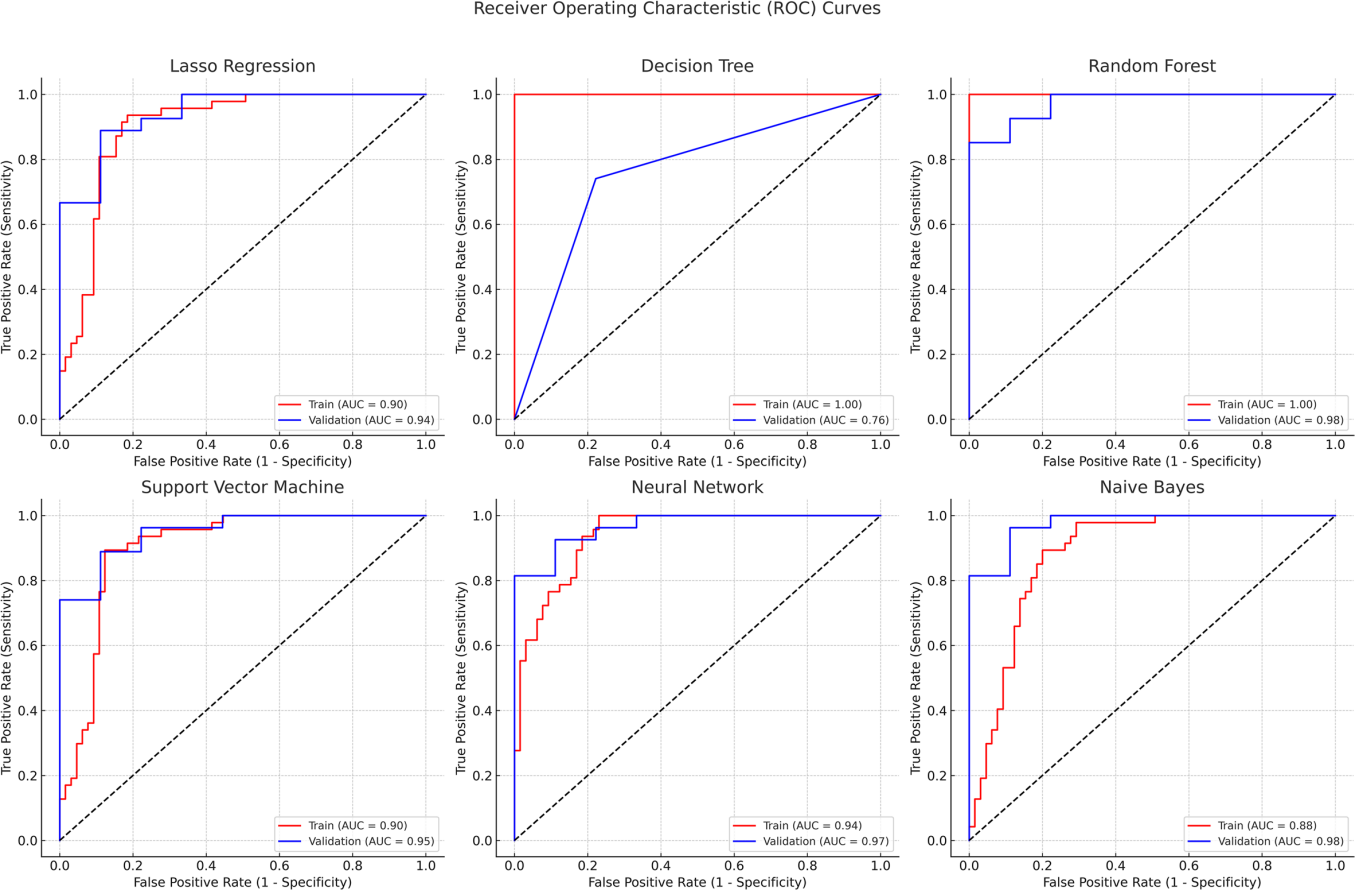
Comparative Analysis of ROC Curves from Multiple Machine Learning Models. ROC curves for six different machine learning models: Lasso Regression, Decision Tree, Random Forest, Support Vector Machine, Neural Network, and Naive Bayes. The area under the curve (AUC) metric was used to evaluate the performance of each model, with a higher AUC indicating better performance.

The employment of machine learning methodologies was directed at determining their predictive efficacy on the dataset. Performance metrics for both the training and validation cohorts, including accuracy, precision, recall, F1-score, and the AUC were computed and are displayed in Tables 3 and 4. The Random Forest notably exhibited superior performance in terms of accuracy and AUC in both cohorts, aligning with findings from past studies on bacterial and viral infections^24^. Meanwhile, ROC curves for Lasso Regression and SVM models suggested a high degree of accuracy in infection type classification. During cross-validation on the training set, the optimized Lasso Regression model attained an AUC score of approximately 0.90. The model's robustness and generalizability were confirmed through its performance on a separate validation set, where it achieved an AUC score of approximately 0.94, demonstrating its ability to effectively distinguish between viral and bacterial infections (Fig. 3).

**Table 3:** Performance metrics of machine learning models on the training cohort.

Model	Accuracy	Precision	Sensitivity	F1 Score	AUC (95% CI)
LR	0.901	0.88	0.928	0.903	0.90 (0.925–0.955)
DT	0.874	0.86	0.891	0.875	1.00 (0.892–0.942)
RF	0.91	0.869	0.964	0.914	1.00 (0.969–0.987)
SVM	0.883	0.839	0.946	0.889	0.90 (0.915–0.947)
NN	0.919	0.871	0.982	0.924	0.94 (0.924–0.982)
NB	0.874	0.86	0.891	0.875	0.88 (0.917–0.950)

*AUC* area under the curve, *CI* confidence interval, *LR* lasso regression, *DT* decision trees, *RF* random forest, *SVM* support vector machine, *NN* neural networks, *NB* naive bayes.

**Table 4:** Performance metrics of machine learning models on the validation cohort.

Model	Accuracy	Precision	Sensitivity	F1 Score	AUC (95% CI)
LR	0.865	0.889	0.843	0.865	0.94 (0.830–0.962)
DT	0.838	0.843	0.843	0.843	0.76 (0.723–0.962)
RF	0.919	0.9	0.948	0.924	0.98 (0.70–0.985)
SVM	0.757	0.679	1	0.809	0.95 (0.797–0.987)
NN	0.919	0.9	0.948	0.924	0.97 (0.650–0.967)
NB	0.811	0.8	0.843	0.821	0.98 (0.756–0.978)

*AUC* area under the curve, *CI* confidence interval, *LR* lasso regression, *DT* decision trees, *RF* random forest, *SVM* support vector machine, *NN* neural networks, *NB* naive bayes.

The feature importance of each variable was evaluated across different machine learning models, revealing NEUp, logMLR, and logPLR to be the significant. The highest importance scores in the Lasso Regression model were found for NEUp (2.0110), logMPVLR (1.0451), and logPLR (0.6210). In the Decision Tree model, a high importance score was assigned to the NEUp variable (0.5127). Notably, the Random Forest model showed elevated scores for NEUp (0.3024) and LYMp (0.1349). The SVM model indicated WBC (0.0528) and NEU (0.0722) as most important, while in the Neural Network model, logMPVLR (0.1306) had the highest score. In the Naive Bayes model, the WBC variable scored slightly higher (0.0556), underscoring their potential utility in diagnostic algorithms (Table 5).

**Table 5:** Feature importance across multiple machine learning models in differentiating bacterial and viral infections.

Model	WBC	NEU	NEUp	LYM	LYMp	logMLR	logNLR	logPLR	logMPVLR
LR	0	0.0622	2.011	0.3163	0.0668	0.4512	0	0.621	1.0451
DT	0.0183	0.1276	0.5127	0.0728	0.08	0.0559	0.1019	0.0308	0
RF	0.04	0.0764	0.3024	0.0989	0.1349	0.0691	0.1486	0.0644	0.0653
SVM	0.0528	0.0722	0.0778	0.0194	0.0333	0.0222	0.0417	0.0167	0.0056
NN	0.0417	0.0361	0.0722	0.025	0.0139	0.0139	0.0583	0.0361	0.1306
NB	0.0556	0.0611	0.0528	0.0194	0.0694	0.0306	0.0417	0.0028	0

*LR* lasso regression, *DT* decision trees, *RF* random forest, *SVM* support vector machine, *NN* neural networks, *NB* naive bayes.

These values represent how much each feature contributes to the model's predictions. The larger the value, the more important the feature is.

**Figure 4:**
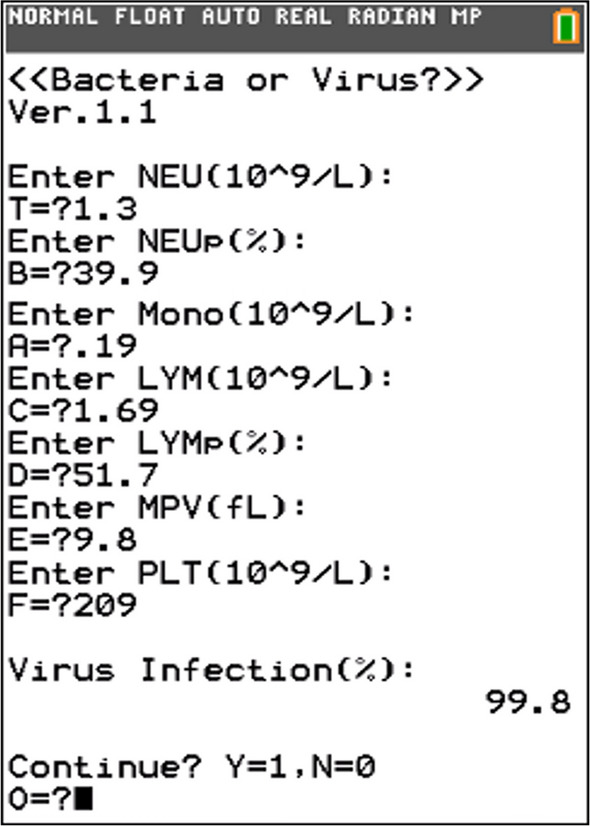
Screenshot of the Lasso Regression Model Program on a TI-84 Calculator. This figure presents a screenshot of the TI-84 calculator running the developed Lasso regression model program. The program enables the user to input five laboratory parameters: Monocytes (MONO), Neutrophils percentage (NEUp), Lymphocytes (LYM), Lymphocytes percentage (LYMp), Platelets (PLT), and Mean Platelet Volume (MPV). The calculator subsequently generates the probability of the infection type, aiding in distinguishing between bacterial and viral infections.

Following deployment, the Lasso Regression model exhibited substantial adeptness in differentiating between bacterial and viral infections. By simply inputting the selected laboratory parameters into the TI-84 calculator, healthcare professionals could expeditiously generate infection probability outcomes (Fig. 4). The model was stringently assessed. The validation cohort in our study, included data from 37 patients (19 bacterial, 18 viral infections). The consistent and effective performance emphasizes the model's robustness and reliability.

## Discussion

This study has highlighted hematological disparities between bacterial and viral infections, shedding light on the pronounced inflammatory response elicited predominantly by bacterial infections. The hematological parameters, MLR, NLR, PLR, and MPVLR have been emphasized as notable biomarkers^25,26^. In line with established literature, viral infections are typically characterized by augmented RBC counts and HGB levels, while bacterial infections are more likely to display heightened WBC, NEU, and MONO counts^27^ (Table 1).

The distinct variations in key hematological parameters such as NEU, NEUp, LYMp, logMLR, and logNLR underscore the differential immunological responses between bacterial and viral infections (Table 2). It is well-established that neutrophils are the primary leukocytes engaged in immune responses during bacterial infections, while lymphocyte-mediated immune responses are predominantly observed during viral infections^28^. The substantial feature importance score of logMLR across various machine learning models accentuates its critical role as a distinguishing hematological parameter, potentially aiding in the enhanced diagnostic differentiation between bacterial and viral infections in our study. Nevertheless, the diagnostic quandaries stemming from the overlapping distributions of WBC and NEU, as illustrated in Fig. 2, emphasize the imperative for a broader diagnostic strategy, transcending the reliance on singular markers^29^.

From a computational viewpoint, the Random Forest emerged as the most proficient predictor for classifying infection types, albeit with Neural Networks showing close prowess^30^. Conversely, SVM and Naive Bayes showcased diverse performances, underscoring the imperative nature of meticulous model selection tailored to specific data characteristics^31^ (Tables 3 and 4). Both Lasso Regression and Random Forest were proficient in differentiating bacterial from viral infections (Fig. 3).

In this study, Lasso Regression was utilized to create a diagnostic model for classifying infection types. The choice of Lasso Regression was predicated on its unique characteristics, which encompass both variable selection and regularization functionalities^32^. This makes it particularly suitable for this type of problem. Although more complex machine learning methodologies are available, Lasso Regression establishes a balance between model intricacy and interpretability^33^. This equilibrium is essential in clinical environments where elucidating the relationship between predictors and outcomes is as vital as achieving prediction accuracy^34,35^.

A noteworthy innovation of this work is the successful amalgamation of the Lasso model with a widely accessible computational tool, the TI-84 calculator. Although both Random Forest and Lasso Regression exhibited commendable performance in our analysis, the computational parsimony of Lasso Regression rendered it a more pragmatic choice for the TI-84 calculator, known for its ease of use, cost-effectiveness, and ubiquitous availability. This integration facilitated the rapid and efficient input of pivotal laboratory parameters including NEU, MONO, NEUp, LYM, LYMp, PLT, and MPV, consequently generating the probability of infection type. By merely inputting the selected laboratory parameters into the calculator, healthcare professionals could promptly ascertain the likelihood of bacterial or viral infections (Fig. 4). No commercial reagents or specific equipment were required in this methodology, promoting its cost-effectiveness and widespread accessibility.

Integral to this discussion is the concept of antibiotic stewardship. Given the emerging global challenge of antibiotic resistance, it is imperative to differentiate bacterial from viral infections to ensure judicious antibiotic use^36^. These findings contribute significantly to antibiotic stewardship efforts by pinpointing potential biomarkers that might expedite accurate diagnosis, thereby minimizing unwarranted antibiotic prescriptions. Emphasizing the need for precise diagnosis and targeted therapies, this study underlines the importance of combining clinical, laboratory, and computational tools in the era of personalized medicine and antibiotic stewardship^37^.

The prospect of amplifying diagnostic precision through the amalgamation of optimization algorithms with machine learning methodologies is indeed exhilarating. Esteemed optimization algorithms such as the refined Grey Wolf Optimizer (LGWO)^38^, Hunger Games Search (HGS)^39^, Shrimp and Goby Association Search algorithm (SGA)^40^, Planet Optimization Algorithm (P.O.A.)^41^, and Runge Kutta optimizer (RUN)^42^ possess the potential to significantly enhance model efficacy. Although the current study did not delve into these optimization techniques, the future incorporation of such advanced optimization algorithms to refine the machine learning models utilized in this study is a significant direction we plan to pursue.

### Limitations

This study acknowledges several limitations. The dataset, while comprehensive, encapsulates a specific patient population with unique characteristics that may influence the performance of the machine learning models. Potential confounding variables, including underlying health conditions and medication usage, were not rigorously controlled, possibly subtly impacting the outcomes. The generalizability of the findings may be contingent on the specific patient population from which the dataset was derived. Despite these considerations, the insights derived from this study are valuable, laying a groundwork for more exhaustive future investigations.

## Conclusion

This study underscores the clinical necessity of accurately and swiftly distinguishing between bacterial and viral pharyngitis. By integrating traditional laboratory techniques with advanced machine learning, a new dimension to the diagnostic potential of hematological markers such as MLR was explored. The notable efficacy of the Random Forest and Lasso Regression in data prediction for this specific dataset suggests that exploring various machine learning techniques could hold promise for further diagnostic advancements.

The adaptation of a Lasso Regression model for use in a TI-84 calculator showcased a practical application of machine learning in clinical settings, enhancing accessibility and ease of use compared to traditional nomograms. These findings illuminate hematological distinctions between viral and bacterial infections in adult patients with pharyngitis, offering MLR as a potential addition to diagnostic methodologies. This not only has the potential to enhance diagnostic accuracy but also to refine therapeutic interventions.

It would be beneficial to extend the application of this model to other types of infections, and to integrate more variables and machine learning techniques, thereby enhancing its utility in infectious disease diagnosis. The results from this study mark a step towards more precise and timely diagnosis of pharyngitis, contributing to better management and treatment of this common condition.

## Data Availability

In adherence to privacy regulations and to ensure the confidentiality of sensitive patient information, the datasets utilized in this study are not publicly available. The research project and access to health databases are overseen by the Ethical Committee of Hebei Provincial Hospital of Traditional Chinese Medicine, which has set guidelines recommending against public dissemination of these datasets. Should researchers wish to request access to the data for academic purposes, they may contact the Corresponding Author who will facilitate the request in accordance with the guidelines set forth by the Ethical Committee of Hebei Provincial Hospital of Traditional Chinese Medicine.

## Abbreviations

GABHS: Group A β-hemolytic streptococcus
CBC: Complete blood count
RBC: Red blood cell count
HGB: Hemoglobin concentration
WBC: White blood cell count
NEU: Neutrophil count
NEUp: Percentage of neutrophils
MONO: Monocyte count
MONOp: Percentage of monocytes
LYM: Lymphocyte count
LYMp: Percentage of lymphocytes
PLT: Platelet count
MPV: Mean platelet volume
ML: Machine learning
MLR: Monocyte to lymphocyte ratio
NLR: Neutrophil to lymphocyte ratio
PLR: Platelet to lymphocyte ratio
MPVLR: Mean platelet volume to lymphocyte ratio
IQR: Interquartile range
SD: Standard deviation
AUC: Area under the curve
CI: Confidence interval
LR: Lasso regression
DT: Decision trees
SVM: Support vector machine
NN: Neural networks
NB: Naive bayes
